# Comparison of cyclic fatigue resistance of XP-endo Shaper, HyFlex CM, FlexMaster and Race instruments

**DOI:** 10.15171/joddd.2018.032

**Published:** 2018-09-18

**Authors:** Mehmet Adiguzel, Ipek Isken, Ismail Ilker Pamukcu

**Affiliations:** Department of Endodontics, Faculty of Dentistry, Mustafa Kemal University, Hatay, Turkey

**Keywords:** Body temperature, cyclic fatigue, FlexMaster, HyFlex CM, Race, XP-endo Shaper

## Abstract

***Background.*** The aim of this study was to compare the cyclic fatigue resistance of XP-endo Shaper, HyFlex CM, FlexMaster and Race rotary instruments at body temperature (37±1°C).

***Methods.*** Twenty XP-endo Shaper (#30/.01), 20 HyFlex CM (#30/.04), 20 FlexMaster (#30/.04) and 20 Race (#30/.04) instruments were tested at body temperature (n=20). The instruments were evaluated in artificial canals with a 3-mm radius of curvature and 60° angle of curvature to the center of the 1.5-mm-wide canal. Each instrument was rotated until fracture occurred and the number of cycles to failure (NCF) recorded. Data were analyzed using one-way ANOVA and Tukey HSD tests (P<0.05).

***Results.*** The difference in the NCF of all the instruments was statistically significant (P<0.05). The order of the instruments from the highest to the lowest NCF was as follows: XP-endo Shaper (3064.0±248.1), HyFlex CM (1120.5±106.1), FlexMaster (569.8±48.4) and Race (445.5±53.5).

***Conclusion.*** Under the limitations of the present study, XP-endo Shaper instruments were more resistant to cyclic fatigue than the #30/.04 nickel-titanium rotary instruments immersed in water at simulated body temperature.

## Introduction

The development of nickel-titanium (Ni-Ti) instruments has ushered in a new era in root canal treatment on account of their high flexibility. However, in spite of the improved strength and flexibility of the Ni-Ti endodontic instruments, there is still a risk of clinicians experiencing instrument fractures during treatment.^1^ Torsional or cyclic fatigue has been identified as one of the reasons for these fractures.^2,3^ Torsional fatigue happens when the ultimate shear strength of an instrument is approached.^4^ Cyclic fatigue happens when tension‒compression stress cycles exceed the maximum flexure capacity while the instrument rotates within a curvature, eventually leading to fracture.^5,6^ The thermomechanical processing and alloying treatments in Ni-Ti alloys improved the superelasticity and the cyclic fatigue resistance of Ni-Ti-based instruments.

Recently, Xp-endo Shaper (FKG Dentaire SA, La Chaux-de-Fonds, Switzerland) systems have been introduced that made use of Ni-Ti MaxWire alloy as XP-Endo Finisher (FKG Dentaire SA). Because of this special alloy, this instrument is soft in its martensitic phase, which is reached at room temperature. However, when placed in the canal at body temperature, it returns to the memorized shape (austenitic phase).^7^ XP-endo Shaper uses a specific geometry with six cutting edges at the apex. With an initial taper of 1%, the XP-endo Shaper file transforms from a straight shape to a serpentine shape. This serpentine shape pushes out the envelope of movement and file reaching a taper of at least 4%. The manufacturer has stated that the technology design provides superelasticity and extreme flexibility. HyFlex (Coltene, Allstatten, Switzerland) rotary instruments are produced from a controlled memory (CM) Ni-Ti wire, which is manufactured using a distinctive process that inspects the material’s memory.^8,9^ FlexMaster (VDW, Munich, Germany) instruments have round, passive tips and triangular cross-section with no radial lands.^9^ Race (FKG Dentaire SA) instruments have a triangular cross-section with alternating cutting edges and a specific anti-screw-in design.^10^

There have been numerous studies on the effects of cyclic fatigue resistance upon the performance and mechanical properties of endodontic instruments.^11,12^ These studies allowed for comparisons between the instruments tested in each experimental design. Lately, researchers revealed that martensitic instruments that are more fatigue resistant than austenitic instruments.^1,13,14^ However, the majority of previous studies were carried out at room temperature, which is considered to change Ni-Ti features.^15-17^ Newer alloys have temperatures close to body temperature, at which the alloy transforms.^14^ Also, in vivo root canal temperature is kept as close as possible to body temperature and canal irrigation agents reach body temperature in a short period of time.^18^ This temperature is higher than those of the conventional austenitic materials used in previous rotary instruments.^1^

The aim of this study was to test the cyclic fatigue resistance of XP-endo Shaper, HyFlex CM, FlexMaster and Race instruments during immersion in water at body temperature. The null hypothesis of the present study was that there would be no difference in cyclic fatigue resistance between the groups.

## Methods

Twenty XP-endo Shaper (#30/.01), 20 HyFlex CM (#30/.04), 20 FlexMaster (#30/.04) and 20 Race (#30/.04) instruments were tested at body temperature (37±1°C) (n=20). Each instrument was examined under a dental operating microscope to define defects or deformities prior to the experiment; none were discarded.

Stainless-steel artificial canals with a 3-mm radius of curvature and 60° angle of curvature to the center of the 1.5-mm-wide canal were prepared in a metal block, as described previously by Larsen et al.^19^ The cyclic fatigue resistance of Ni-Ti rotary files was tested using a static model. A metal block was placed on a plastic container; 200 mL of deionized water at 37°C was then prepared and placed in a plastic container. To maintain the desired temperatures and volume, the water was refreshed (added-removed) when a new instrument was changed. The study model in the container was allowed to equilibrate temperatures. The temperature was measured with a thermometer. The device and the plastic container with the metal block on it were positioned and fixed. The instruments were operated using a torque-controlled motor (Silver; VDW, Munich, Germany). No torque limit was applied; revolutions per minute (rpm) were regulated according to each manufacturer’s recommendations (XP-endo Shaper; 800 rpm, HyFlex CM; 500 rpm, FlexMaster; 280 rpm and Race; 600 rpm). The insertion depth was standardized to 19 mm for all the instruments. The metal block was covered with a glass plate to prevent the instruments from slipping out during the test. The instruments were rotated in water at body temperature until fracture occurred, and the number of the times to failure was recorded. The number of cycles to fracture (NCF) was then calculated by using the following formula:

NCF = time (seconds) to failure × rotational speed/60

The length of the fractured tip was measured using a digital caliper (Digimatic, Mitutoyo Co., Kawasaki, Japan).

### Statistical Analysis

Data were analyzed with SPSS 21.0 (SPSS Inc., Chicago, IL). Normal distribution of the variables was tested using Kolmogorov–Smirnov test. Data were analyzed using one-way ANOVA and Tukey HSD tests to determine any statistical difference between the groups. Statistical significance was set at P < 0.05.

### Scanning Electron Microscopic Analysis

The fractured instruments were evaluated under a scanning electron microscope (SEM). SEM imaging was used to take pictures of the fractured surfaces of the representative samples.

## Results

**Figure 1:**
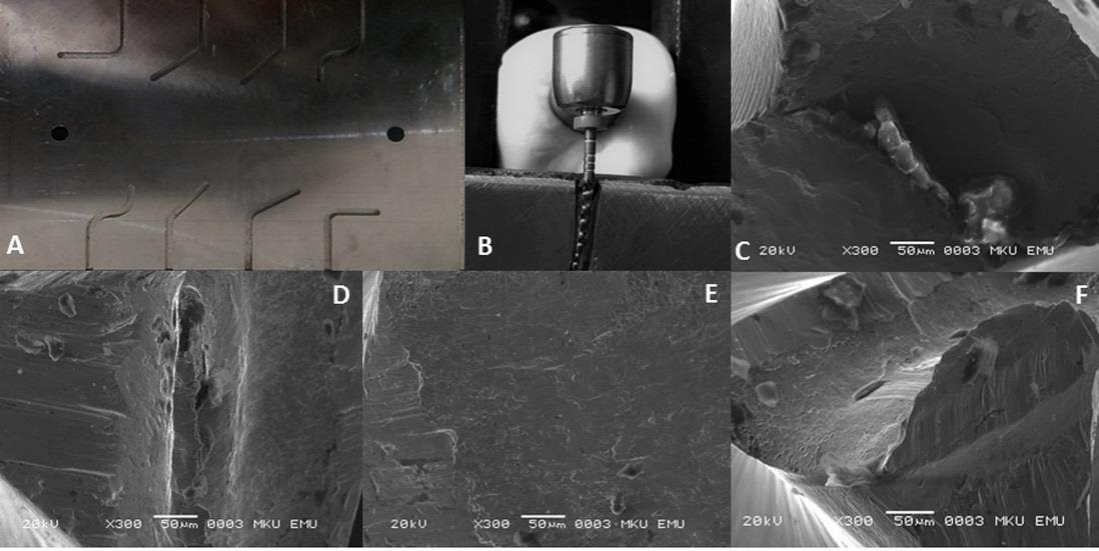
(A) Photograph of canal metal block. (B) Photograph of cyclic testing device used for the fatigue test. Representative SEM appearances of the (C) Race, (D) HyFlex CM, (E) FlexMaster and (F) Xp-endo Shaper files after cyclic fatigue testing (×300, original magnification).

The results for the XP-endo Shaper (3064.0±248.1), HyFlex CM (1120.5±106.1), FlexMaster (569.8±48.4) and Race (445.5±53.5) at body temperature are shown in Table 1. There were significant differences between the cyclic fatigue resistances of instruments (P<0.05). The XP-endo Shaper demonstrated a higher level of cyclic fatigue resistance than the #30/.04 Ni-Ti rotary systems (P<0.05). The order of the instruments from the highest to the lowest NCF was as follows: XP-endo Shaper>HyFlex CM>FlexMaster>Race (P<0.05). The length of the fractured part of the XP-endo Shaper exhibited a higher mean than others (P<0.05). However, there was no significant difference in the length of the fractured parts of other groups (P>0.05). The SEM images of the fractured surface indicated the character of the mechanical damage of the cyclic fatigue failure in all the groups (Figure 1). SEM analysis of the representative samples showed crater-like formations along with dimples and microbubbles.

**Table 1:** Mean values (± standard deviations) for the NCF and fragment length

**Groups**	**No.**		**NCF**			**Fragment length**	
**P-Endo Shaper**	20	3064.0	±	248.1^a^	6.14	±	0.33^a^
**HyFlex CM**	20	1120.5	±	106.1^b^	5.03	±	0.24^b^
**FlexMaster**	20	569.8	±	48.4^c^	5.26	±	0.28^b^
**Race**	20	445.5	±	53.5^d^	5.18	±	0.38^b^

Different superscript letters show a significant difference between groups. (P < 0.05)

## Discussion

Selecting an instrument with better fracture resistance could reduce instrument fracture in clinical situations.^15-17^ Lower-tapered files ensure fewer file fractures and a more conservatively shaped canal.^17^ Taper is one of the factors having an effect on mechanical properties of Ni-Ti files because file diameter is inversely proportional to cyclic fatigue resistance.^17^ In this study, because of the above-mentioned reasons, #30/.04 instruments were selected. Although taper of XP-endo Shaper files were initially 30/.01, the manufacturer claims that the instruments’ swaggering motion leads to a taper expansion up to 30/.04; therefore, in the present study, the nickel-titanium rotary instruments that were compared had 30/.04 tapers.

The present study assessed the cyclic fatigue resistance of XP-endo Shaper, HyFlex CM, FlexMaster and Race instruments at body temperature. The results demonstrated that the cyclic fatigue resistance of XP-endo Shaper instruments was superior to that of the corresponding #30/.04 Ni-Ti rotary system instruments. Thus, the null hypothesis of no difference in the cyclic fatigue resistance of the tested systems was rejected.

Currently, there is limited knowledge in the literature that has evaluated XP-endo Shaper in cyclic fatigue performance at body temperature or room temperature.^20,21^ Recently, Elnaghy et al^20^ compared the cyclic fatigue resistance of the XP-endo Shaper instruments with different NiTi alloy instruments at body temperature. The results were that XP-endo Shaper instruments exhibited the highest cyclic fatigue resistance between the tested instruments, consistent with results of the present study. XP-endo Shaper exhibited higher cyclic fatigue resistance than other systems because of its changing taper. The initial small ISO diameter and narrow taper make the instrument more resistant to cyclic fatigue.^20^ Also, the XP-endo Shaper instruments have superelasticity and shape memory because of the exclusive MaxWire alloy. These instruments are able to behave according to the temperature. Their shape is predetermined for body temperature.^20^

In the present study, XP-endo Shaper and HyFlex CM instruments' resistance to cyclic fatigue values were found to be higher when compared with FlexMaster and Race instruments. This result might be attributed to the metallurgical differences among the instruments. XP-endo Shaper and HyFlex CM instruments are produced from heat-treated alloys, whereas FlexMaster and Race instruments are produced from conventional NiTi alloy.^20^ Instruments made from conventional NiTi alloy display an austenitic phase at room temperature, whereas heat-treated alloys, in addition to the austenite, also include martensite and R-phase.^22^ FlexMaster and Race instruments are in austenic phase with Af temperature below body temperature. The Af temperature of Hyflex CM was about 47°C, indicating that this file will have a mixed martensitic R-phase and austenitic structure at body temperature.,^23^ The transition of XP-endo Shaper instrument from the martensite phase to the austenite phase takes place at body temperature between 32°C and 37°C with Af temperature around 35°C.^24^ These features of XP-endo Shaper and HyFlex CM instruments might have positively affected the cyclic fatigue resistance because the experiment was carried out at body temperature.

de Vasconcelos et al^1^ compared the effect of HyFlex CM systems on cyclic fatigue resistance at body temperature with that of other systems. The results showed that HyFlex CM and Vortex Blue had better fatigue resistance than ProTaper Universal and TRUShape. Immersion of the instruments in water at simulated body temperature was related to a significant decline in the cyclic fatigue resistance of the instruments.^1^ The researchers found that martensitic instruments are more flexible and more fatigue resistant than austenitic instruments.^1,13,14^ It has also been discovered that in vivo intracanal temperature range is 31–35°C, and that irrigation solutions used in the intracanal turn at body temperature within a short span of time.^1,8,25^ In vitro fatigue testing uncovers a basic similarity to the clinical condition.^1^ Therefore, cyclic fatigue test should be carried out at 37°C to simulate patient body temperature.

In previous studies performed at room temperature, Zhao et al^26^ reported that HyFlex CM (#30/.06) instruments comprised of new heat-treated alloy were more resistant to fatigue failure than Race (#30/.06) instruments. The results of this study are consistent with those of the present study. Instruments produced from CM Wire had a superior fatigue life and higher fracture resistance than the conventional Ni-Ti wire files with identical design.^8^

The SEM images of the fracture surface of groups displayed the character of the mechanical damage of the cyclic fatigue failure. SEM analysis of the representative samples showed crater-like formations along with dimples and microbubbles. These characteristics demonstrated that the instruments had undergone a ductile mode of fracture, which is mostly viewed in cyclic fatigue failure. Also, presence of fatigue striations and absence of circular abrasion showed the flexural fatigue failure.

The fractured part of the XP-endo Shaper displayed a higher mean length than the other instruments. The XP-endo Shaper instrument expansion at body temperature contributed to its helical motion in the canal. It had a “snake” shape. For these reasons, a difference can be considered to be present.

## Conclusions

Under the limitations of the present study, XP-endo Shaper instruments were found to be more resistant to cyclic fatigue than the #30/.04 Ni-Ti rotary instruments immersed in water at simulated body temperature.

## Acknowledgements

None.

## Authors’ contributions

MA was responsible for conception, design, and supervision of this study. MA, II, and IIP performed data collection and processing. MA was also responsible for analysis and interpretation of data. MA, II, and IIP together reviewed the literature and drafted the manuscript. All authors contributed to the critical revision of the manuscript, and have read and approved the final version.

## Competing interests

The authors declare no competing interests with regards to the authorship and/or publication of this article.

## Ethics approval

Not applicable.
